# What is the impact of radical hysterectomy on endometrial cancer with cervical involvement?

**DOI:** 10.1186/s12957-020-01876-x

**Published:** 2020-05-21

**Authors:** Salim Abraham Barquet-Muñoz, David Cantú-de-León, Antonio Bandala-Jacques, Aarón González-Enciso, David Isla-Ortiz, Diddier Prada, Luis A. Herrera, R. A. Salcedo-Hernández

**Affiliations:** 1 Departamento de Ginecología, Instituto Nacional de Cancerología, San Fernando 22, Tlalpan, 14080 Mexico City, Mexico; 2 Unidad de Investigación Biomédica en Cáncer, Instituto Nacional de Cancerología, Mexico City, Mexico; 3 Instituto de Investigaciones Biomédicas, Universidad Nacional Autónoma de México, Mexico City, Mexico; 4 Departamento de Cirugía, Instituto Nacional de Cancerología, Mexico City, Mexico; 5 Departamento de Informática Biomédica, Faculty of Medicine, Universidad Nacional Autónoma de México, Mexico City, Mexico; 6 Instituto Nacional de Medicina Genómica, Mexico City, Mexico

**Keywords:** Endometrial neoplasms, Hysterectomy, Carcinoma, Endometrioid

## Abstract

**Background:**

When endometrial carcinoma invades the cervical stroma, overall survival and disease-free survival decrease. However, it is still controversial whether patients in suspected stage II should be treated with radical hysterectomy. The goal of this study is to describe the role of radical hysterectomy in patients with endometrial carcinoma and cervical involvement.

**Methods:**

This was a retrospective cohort study were a total of 239 patients with endometrial carcinoma with cervical involvement from Mexico City’s National Cancer Institute were divided according to the type of hysterectomy, and the outcomes were compared using statistical analysis.

**Results:**

The 5-year overall survival was 75.76% for the simple hysterectomy group and 89.19% for the radical hysterectomy group, without achieving statistical significance. The 5-year disease-free survival was 72.95% for the simple hysterectomy group and 64.31% for the radical hysterectomy group, without achieving statistical significance. Radicality was associated with longer surgical times, intraoperative complications, and bleeding over 500 ml.

**Conclusions:**

In patients with endometrial carcinoma with cervical involvement, radical hysterectomy does not improve prognosis or alter adjuvant therapy.

## Background

Endometrial carcinoma invades the cervical stroma in 5–10% of cases [1]. When there is exclusive cervical invasion, defined as International Federation of Gynecology and Obstetrics (FIGO) stage II, the 5-year overall survival decreases to 75%, compared to 88% for stage I [2]. It is still controversial whether patients in suspected stage II should be treated with radical hysterectomy [3, 4]. The goals of this surgical management are to obtain an optimal cytoreduction and identify parametrial involvement, with the consequent change in clinical staging, prognosis, and need for adjuvancy [4]. It should be noted that radical hysterectomy carries a risk of complications that could delay adjuvancy [5]. Additionally, cervical involvement could be associated with other poor prognostic factors, such as lymphovascular invasion, unfavorable histologies, deep myometrial invasion, and ovarian and lymph node involvement [2, 6, 7]. This brings to question the therapeutic role of radical hysterectomy in patients with endometrial carcinoma with cervical involvement.

The goal of this study is to describe the role of radical hysterectomy in patients with endometrial carcinoma and cervical involvement, independent of clinical stage.

## Methods

This was a retrospective cohort study with patients treated at Mexico City’s National Cancer Institute between January 2005 and December 2018. The data were obtained from the clinical files of the electronic records of patients who met inclusion criteria, after which a database was created and analyzed.

The inclusion criteria were patients with a diagnosis of endometrial carcinoma with involvement of cervical stroma suspected preoperatively by imaging (computed tomography (CT) or magnetic resonance imaging (MRI)) or by biopsy, confirmed by the Pathology Department, and treated with hysterectomy. Patients not undergoing hysterectomy, those who received neoadjuvant chemotherapy, those with two primary malignancies, and those with insufficient data for analysis were excluded. All hysterectomies were performed by gynecologic oncologists or by oncologic surgeons with experience in gynecologic cancer. For patients requiring some type of adjuvant treatment, the radiotherapy schemes were an external beam radiotherapy regimen of 45 Gy in 25 fractions, high-dose-rate brachytherapy of 24 Gy in 4 sessions, or low-dose-rate brachytherapy from 30 to 35 Gy. For patients who were prescribed chemotherapy, the scheme used more frequently was carboplatin with paclitaxel for 4 to 6 cycles at the discretion of the medical oncologist.

Clinical, surgical, treatment, and pathological variables were identified. A pathology review was performed by an oncologic pathologist. The patients were divided into two groups, the first contained patients undergoing simple hysterectomy and the second contained patients undergoing radical hysterectomy. Overall survival was defined as the time period between diagnosis and death or the date last seen. Disease-free survival was defined as the time period between surgery and recurrence or the date last seen.

For the descriptive analysis, central tendency measures were used. The median and interquartile range were used for continuous variables, and absolute and relative frequencies were used for categorical variables. For the comparative analysis, Wilcoxon’s rank-sum test, chi-squared, or Fisher’s exact test were used depending on the analyzed variable. Logistic regressions were used to obtain odds ratios and establish factors associated with radical hysterectomy. Survival curves were generated with the Kaplan-Meier estimator and compared with the log-rank test. Univariate and multivariate analyses were performed with Cox regression. Statistical significance was defined as a *p* value < 0.05. Statistical analysis was performed with STATA software, version 13.0 (College Station, TX). This study was approved by the Institutional Review Board (Comité de Investigación y Ética) with approval reference CEI051/16.

## Results

Out of 1014 identified patients with endometrial carcinoma, 239 (23.6%) met all inclusion criteria and were included in the analysis. The median age was 55.64 (interquartile range [IQR] 47.2–63.5) years, and the median weight was 67 (IQR 59–79.8) kg. The most frequent histology was endometrioid adenocarcinoma, with 177 (74.06%) cases. The median tumor size was 12 (IQR 5.5–65) mm, and there were 110 (46.03%) grade 2 cases. A total of 165 (74.32%) cases had lymphovascular invasion. Additionally, the median cervical stromal invasion depth was 6 (IQR 3–12.5) mm, with a depth invasion/stromal rate of 0.5 (IQR 0.25–0.92). Pelvic lymphadenectomy was performed on 165 (69%) patients, of which 70 (42.42%) had lymphatic disease. Likewise, 135 (56.5%) patients underwent para-aortic lymphadenectomy, of which 40 (29.63%) had lymph node metastasis. Regarding clinical stage, 98 (41%) patients were diagnosed with stage II disease, and 95 (39.7%) were diagnosed with stage III disease. A total of 182 (76.2%) patients underwent simple hysterectomy, and 39 (16.32%) underwent radical hysterectomy. Out of the patients who received adjuvant treatment, 153 (64.0%) received external beam radiotherapy, 165 (69.0%) received brachytherapy, and 129 (53.97%) received chemotherapy (Table 1). Of this group that received adjuvant treatment, 109 (45.6%) received any type of radiotherapy and chemotherapy, and 97 (40.6%) received only brachytherapy with chemotherapy.

**Table 1:** Comparison according to the type of hysterectomy in patients with endometrial carcinoma with cervical involvement (*N* = 239)

	SH, 200 (83.68%)	RH, 39 (16.32%)	Total, 239	*p*
Age, years^¥^	55.42 (46.9–63.9)	56.55 (48.7–62.4)	55.64 (47.2–63.5)	0.756
Weight, kg^¥^	67 (59.8–79.9)	66 (57–79)	7 (59–79.8)	0.643
BMI^¥^	28.55 (25.2–33.8)	27.83 (24.4–32.5)	28.51 (25.1–33.3)	0.600
Positive cytology^§^	82 (41)	21 (53.85)	103 (43.10)	0.138
**Histology**^**§**^				
**Endometrioid**	**154 (77)**	**23 (58.97)**	**177 (74.06)**	**0.007**
**Serous papillary**	**15 (7.5)**	**6 (16.38)**	**21 (8.79)**	
**Clear cell**	**4 (2.0)**	**1 (2.56)**	**5 (2.09)**	
**Mixed**	**26 (13.0)**	**6 (15.38)**	**32 (13.39)**	
**Carcinosarcoma**	**1 (0.5)**	**3 (7.69)**	**4 (1.67)**	
Tumor size, mm^¥^	15 (5.3–70.0)	5 (6–50)	12 (5.5–65.0)	0.349
Grade^§^				
G1	9 (4.59)	4 (10.26)	13 (5.44)	0.130
G2	96 (48.0)	14 (35.90)	110 (46.03)	
G3	39 (19.50)	5 (12.82)	44 (18.41)	
Poor prognostic histology	56 (28)	16 (41.03)	72 (30.13)	
Lymphovascular invasion^§^	134 (72.43)	31 (83.78)	165 (74.32)	0.183
Myometrial involvement^§^				
Superficial	5 (2.50)	1 (2.56)	6 (2.51)	0.994
< 50%	52 (26.00)	10 (25.64)	62 (25.94)	
> 50%	125 (62.50)	25 (64.10)	150 (62.76)	
NA	18 (9.0)	3 (7.69)	21 (8.79)	
**Depth of invasion, mm**^**¥**^	**5 (3–12)**	**10.5 (5–15)**	**6 (3–12.5)**	**0.005**
**Depth invasion/thickness rate**^**¥**^	**0.48 (0.25–0.67)**	**0.52 (0.4–0.87)**	**0.5 (0.25–0.92)**	**0.028**
Ovarian involvement^§^	35 (17.50)	7 (17.95)	42 (17.57)	0.946
**Parametrial involvement**^**§**^	**9 (4.50)**	**7 (17.95)**	**16 (6.69)**	**0.002**
Uterine serosa involvement^§^	18 (9.0)	5 (12.82)	23 (9.62)	0.459
Pelvic lymph node involvement, *n* = 165^§^	55 (41.04)	15 (48.39)	70 (42.42)	0.456
Para-aortic lymph node involvement, *n* = 135^§^	34 (31.78)	6 (21.43)	40 (29.63)	0.286
Stage^§^				
II	87 (43.50)	11 (28.21)	98 (41.00)	0.124
III	73 (36.50)	22 (56.41)	95 (39.75)	
IV	31 (15.5)	4 (10.26)	35 (14.64)	
NA	9 (4.50)	2 (5.13)	11 (4.60)	
Any radiotherapy^§±^	150 (75.00)	32 (82.05)	182 (76.2)	0.345
Brachytherapy	135 (67.50)	30 (76.92)	165 (69.04)	0.244
External beam radiotherapy	124 (62)	29 (74.36)	153 (64.02)	0.141
Chemotherapy^§^	107 (53.50)	22 (56.41)	129 (53.97)	0.739
Recurrence^§^	46 (23.00)	11 (28.21)	57 (23.85)	0.485
Death^§^	35 (17.50)	5 (12.82)	40 (16.74)	0.474

*SH* simple hysterectomy, *RH* radical hysterectomy, *BMI* body mass index, *NA* not applicable/unavailable

^¥^Median (interquartile range), statistical analysis: sum of ranks

^§^Absolute value (relative analysis), statistical analysis: chi-squared

^±^Low-dose-rate brachytherapy, high-dose-rate brachytherapy, and external beam radiotherapy

In the comparative analysis between the types of hysterectomies, a statistically significant difference was found regarding histology, in which there was a higher percentage of histologies with poor prognosis in the radical hysterectomy group (23% vs. 41%, *p* = 0.007). The radical hysterectomy group had a higher median depth of cervical stromal invasion (10.5 mm [IQR 5–15] vs. 5 mm [IQR 3–12], *p* = 0.005) and a higher depth of invasion/thickness rate (0.52 [IQR 0.4–0.87] vs. 0.48 [IQR 0.25–0.67]) than the simple hysterectomy group. No statistically significant difference was found in the other variables, including histologic grade, lymphovascular invasion, depth of myometrial involvement, lymph node disease, and adjuvancy. Likewise, the percentages of recurrence and death were similar in both groups (Table 1).

Regarding the surgical variables, there were a total of 18 (7.53%) intraoperative complications and 9 (3.77%) reinterventions. The intraoperative complications were as follows: 5 (27.8%) bladder injuries, 5 (27.8%) accidental injuries to a blood vessel, 4 (22.2%) intestinal injuries, 2 (11.1%) ureteral injuries, and 2 (11.11%) injuries to a nerve. Of the reinterventions, 3 (33.33%) were due to bleeding, 2 (22.22%) were due to gastrointestinal perforation, and 4 (44.44%) were due to the presence of accumulated fluid. The surgical variables associated with radical hysterectomy were para-aortic lymphadenectomy (OR 2.21, 95% CI 1.04–4.04, *p* = 0.038), longer surgical procedure duration (OR 1.001, 95% CI 1.0007–1.008, *p* = 0.020), intraoperative complications (OR 2.85, 95% CI 1.01–8.12, *p* = 0.050), and bleeding over 500 ml (OR 2.23, 95% CI 1.07–4.68, *p* = 0.033). There were no differences regarding the need for blood transfusion, need for reintervention, or type of approach (laparoscopic or laparotomy) (Table 2).

**Table 2:** Surgical variables according to the type of hysterectomy in patients with endometrial carcinoma with cervical involvement (*N* = 239)

	Total	SH, 200 (83.68)	RH, 39 (16.32)	OR	95% CI	*p*
Pelvic lymphadenectomy^§^	165 (69.04)	134 (67.00)	31 (79.49)	1.91	0.83–4.38	0.128
**Para-aortic lymphadenectomy**^**§**^	**135 (56.49)**	**107 (53.50)**	**28 (71.79)**	**2.21**	**1.04–4.069**	**0.038**
**Surgical time, min**^**¥**^	**210.5 (160–295)**	**210 (152.5–280)**	**238 (205–335)**	**1.001**	**1.0007–1.008**	**0.020**
**Intraoperative complications**^**§**^	**18 (7.53)**	**12 (6.00)**	**6 (15.38)**	**2.85**	**1.01–8.12**	**0.050**
Surgical bleeding, ml^¥^	350 (200–550)	350 (200–500)	400 (300–1000)	1.003	0.99–1.0001	0.165
**Bleeding > 500 ml**^**§**^	**55 (25.58)**	**40 (22.60)**	**15 (39.47)**	**2.23**	**1.07–4.68**	**0.033**
Blood transfusion^§^	65 (27.19)	50 (25)	15 (38.46)	0.999	0.99–1.00	0.136
Reintervention^§^	9 (3.77)	7 (3.50)	2 (5.13)	1.49	0.29–7.46	0.627
Laparoscopy^§^	15 (6.28)	13 (6.5)	2 (5.13)	0.77	0.168–3.59	0.747

*SH* simple hysterectomy, *RH* radical hysterectomy, *OR* odds ratio, *95% CI* 95% confidence interval

^¥^Median (interquartile range)

^§^Absolute value (relative analysis)

**Fig. 1:**
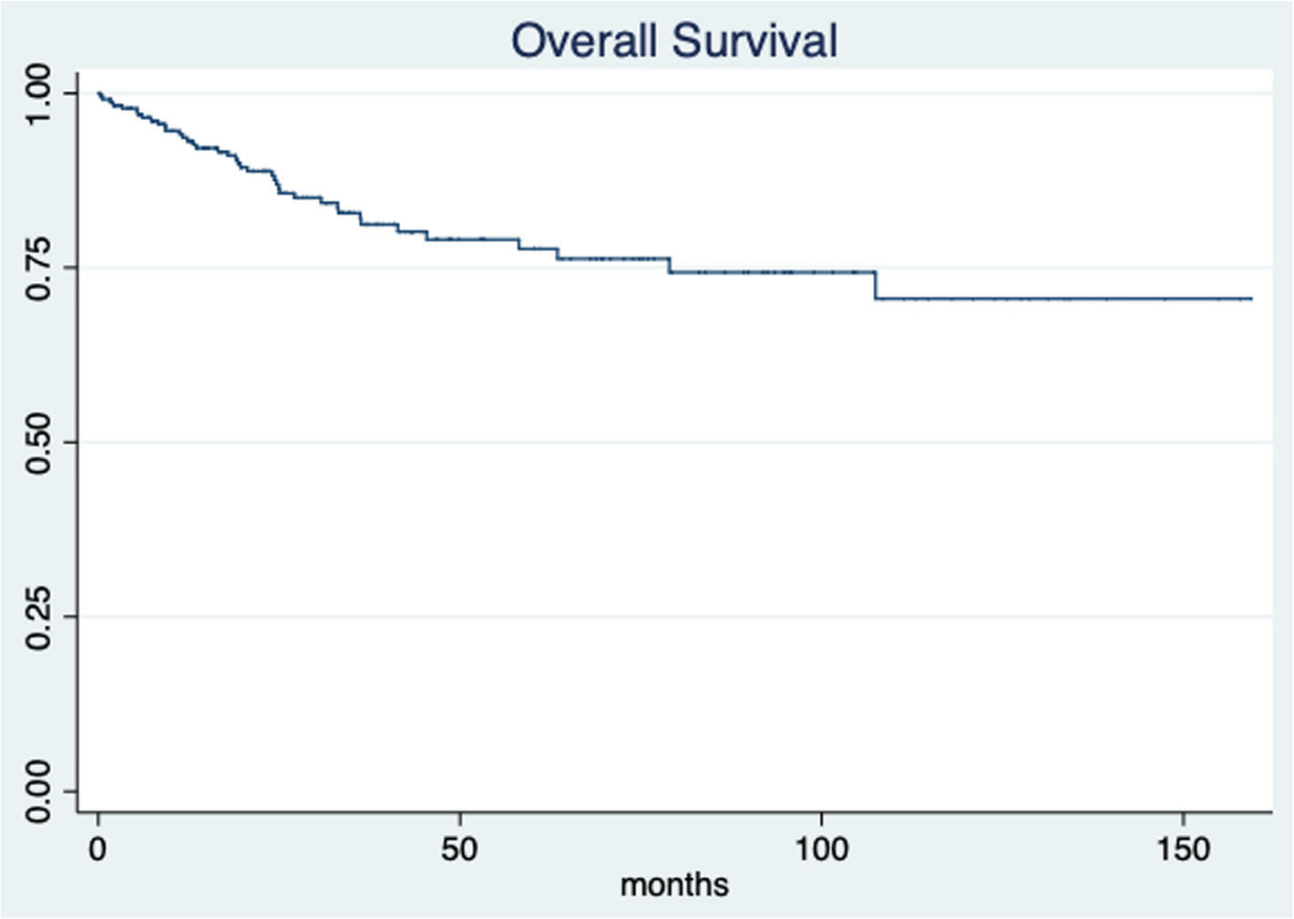
Overall survival rate for all patients

**Fig. 2:**
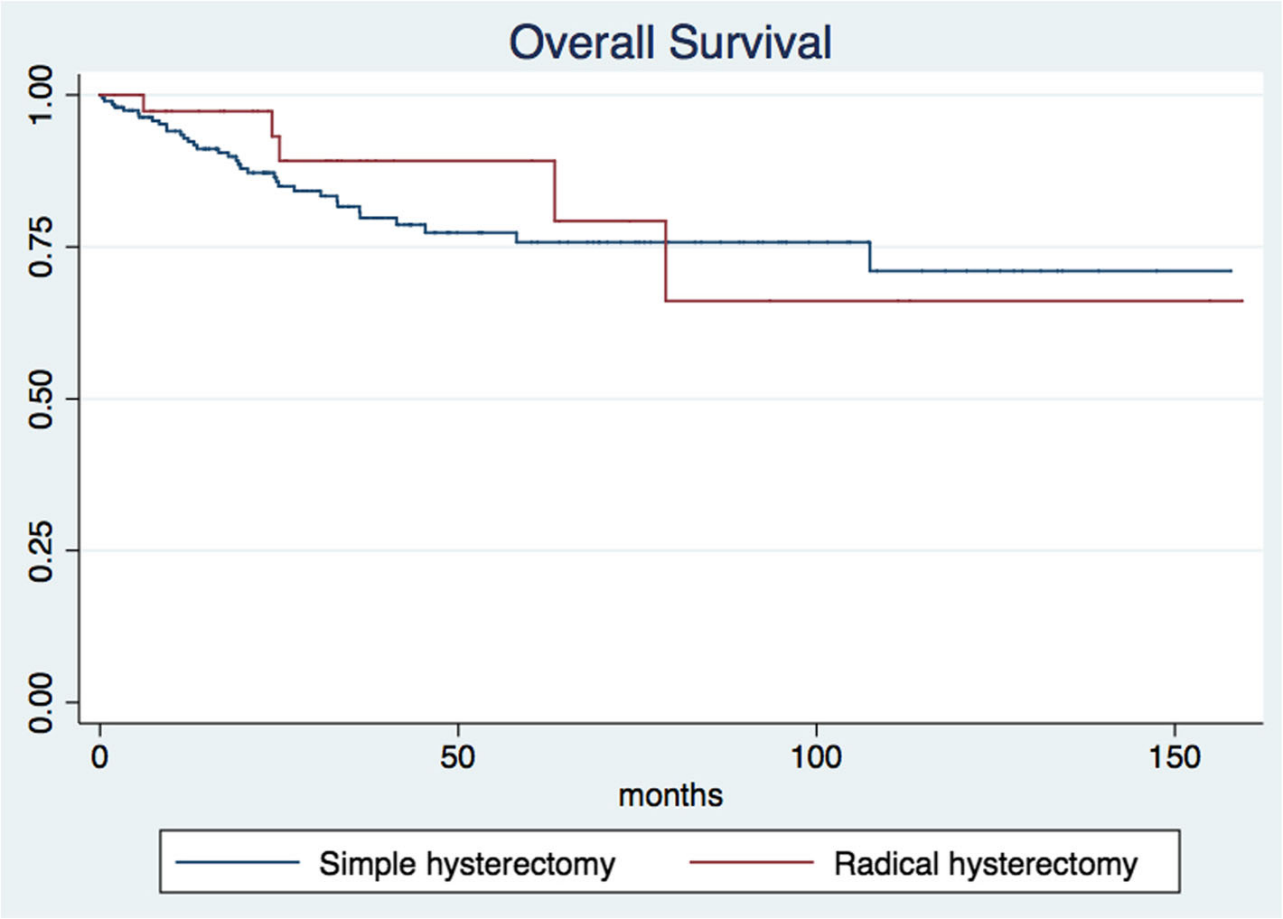
Overall survival rates of the simple and radical hysterectomy groups (*p* = 0.513)

**Fig. 3:**
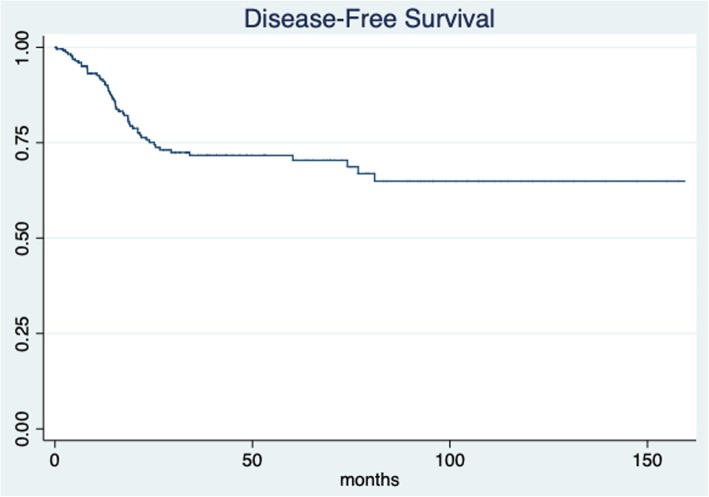
Disease-free survival rate for all patients

**Fig. 4:**
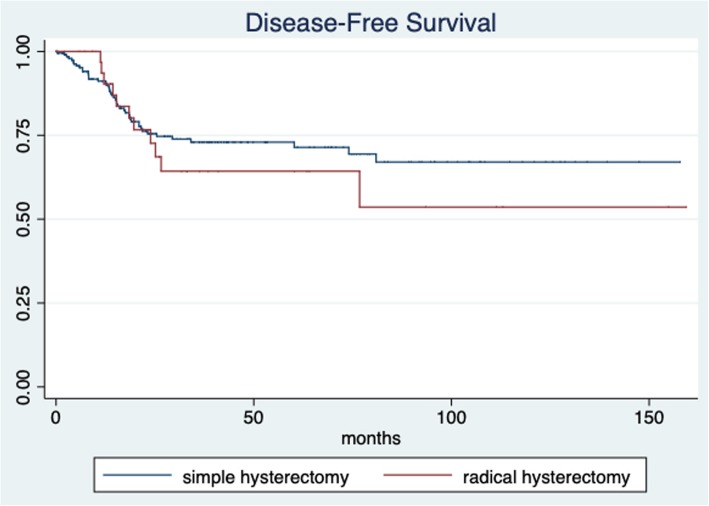
Disease-free survival rates of the simple and radical hysterectomy groups (*p* = 0.534)

The median patient follow-up time was 30.8 months (IQR 14.74–53.37; min 1.63 and max 159.4 months). The 5-year overall survival for all patients was 77.69% (95% CI 69.9–83.7) (Fig. 1). The 5-year overall survival was 75.76% (95% CI 67.03–82.47) for the simple hysterectomy group and 89.19% (95% CI 69.57–96.46) for the radical hysterectomy group, without achieving statistical significance (*p* = 0.513) (Fig. 2). The 5-year disease-free survival for the whole group was 71.65% (95% CI 64.44–76.66) (Fig. 3). The five-year disease-free survival was 72.95% (95% CI 65.16–79.28) for the simple hysterectomy group and 64.31% (95% CI 43.41–79.17) for the radical hysterectomy group, without achieving statistical significance (*p* = 0.534) (Fig. 4).

In the multivariate analysis, the factors independently associated with overall survival (Table 3) were the presence of grade 3 or poor prognostic histology (HR 3.39, 95% CI 1.16–9.92, *p* = 0.026), receiving radiotherapy (HR 0.12, 95% CI 0.004–0.39, *p* < 0.001), and receiving chemotherapy (HR 3.74, 95% CI 1.16–12.09, *p* = 0.027). For disease-free survival (Table 4), the multivariate analysis found independent associations for tumor size (HR 0.98, 95% CI 0.96–0.99, *p* = 0.031) and receiving radiotherapy (HR 0.12, 95% CI 0.04–0.39, *p* < 0.001). In the univariate analysis, radical hysterectomy was not associated with overall survival (HR 0.73, 95% CI 0.29–1.87, *p* = 0.515) or disease-free survival (HR 1.23, 95% CI 0.64–2.38, *p* = 0.535).

**Table 3:** Prognostic factors associated with overall survival in patients with endometrial carcinoma with cervical involvement (*N* = 239)

	Univariate			Multivariate		
	HR	95% CI	*p*	HR	95% CI	*p*
Age	1.01	0.98–1.04	0.461			
Age > 45 years	1.57	0.69–3.55	0.284			
BMI	1.01	0.95–1.06	0.717			
Obesity (BMI > 30)	1.21	0.64–2.28	0.560			
BPH	**2.34**	**1.25**–**4.43**	**< 0.001**	0.94	0.38–2.26	0.887
Tumor size, mm	0.99	0.98–1.001	0.587			
Tumor size > 4 cm	1.01	0.38–2.83	0.991			
**Grade 1-2 vs. 3-BPH**	**4.49**	**2.19**–**9.22**	**< 0.001**	**3.39**	**1.16**–**9.92**	**0.026**
LVI	**2.18**	**1.15**–**4.12**	**0.017**	0.52	0.21–1.27	0.152
Myometrial involvement >50%	**2.54**	**1.12**–**5.79**	**0.026**	0.97	0.35–2.68	0.958
Depth of invasion	1.01	0.97–1.05	0.660			
Depth invasion/thickness rate	0.70	0.15–3.18	0.646			
Ovarian involvement	1.69	0.83–3.47	0.148			
Parametrial involvement	1.61	0.49–5.22	0.432			
Uterine serosa involvement	1.43	0.51–4.02	0.495			
Pelvic node involvement	**2.03**	**0.96**–**4.29**	**0.064**	1.16	0.48–2.78	0.740
Para-aortic node involvement	1.87	0.82–4.28	0.136			
Distant disease	**2.38**	**1.04**–**5.45**	**0.039**	0.98	0.31–3.18	0.986
Radical hysterectomy	0.73	0.29–1.87	0.515			
**Radiotherapy**	**0.32**	**0.16**–**0.63**	**0.001**	**0.12**	**0.04**–**0.39**	**< 0.001**
**Chemotherapy**	**2.06**	**1.04**–**4.07**	**0.037**	**3.74**	**1.16**–**12.09**	**0.027**

*HR* hazard ratio, *95% CI* 95% confidence interval, *BMI* body mass index, *BPH* bad prognostic histology (serous papillary, clear cell, mixed, carcinosarcoma), *LVI* lymphovascular invasion

**Table 4:** Prognostic factors associated with disease-free survival in patients with endometrial carcinoma with cervical affection (*N* = 239)

	Univariate			Multivariate		
	HR	95% CI	*p*	HR	95% CI	*p*
**Age**	**1.03**	**1.01–1.05**	**0.012**			
**Age > 45 years**	**2.91**	**1.24–6.81**	**0.014**	5.50	0.94**–**32.00	0.060
BMI	1.02	0.97**–**1.06	0.578			
Obesity (BMI > 30)	1.29	0.78**–**2.18	0.350			
**BPH**	**2.47**	**1.45–4.19**	**0.001**	0.89	0.24**–**3.31	0.867
**Tumor size, mm**	**0.99**	**0.98–0.99**	**0.047**	**0.98**	**0.96–0.99**	**0.031**
Tumor size > 4 cm	0.72	0.34**–**1.53	0.395			
**Grade 1–2 vs. 3–BPH**	**3.01**	**1.73–5.23**	**< 0.001**	2.92	0.62**–**13.64	0.171
**LVI**	**2.62**	**1.51–4.52**	**0.001**	0.38	0.98**–**1.53	0.98
**Myometrial involvement > 50%**	**2.33**	**1.20–4.52**	**0.012**	2.64	0.52**–**13.33	0.239
Depth of invasion	0.97	0.92**–**1.02	0.238			
Depth invasion/thickness rate	0.77	0.24**–**2.49	0.658			
**Ovarian involvement**	**2.67**	**1.53–4.67**	**0.001**	0.61	0.14**–**2.61	0.503
**Parametrial involvement**	**2.91**	**1.24–6.83**	**0.014**	13.20	0.78**–**222.04	0.073
**Uterine serosa involvement**	**2.05**	**0.92–4.54**	**0.077**	0.13	0.001**–**1.44	0.096
**Pelvic node involvement**	**1.66**	**0.88–3.12**	**0.112**	2.23	0.56**–**8.84	0.253
**Para-aortic node involvement**	**1.73**	**0.88–3.39**	**0.110**	1.67	0.47**–**5.93	0.426
**Distant disease**	**4.90**	**2.61–9.21**	**< 0.001**	1.22	0.28**–**5.32	0.783
Radical hysterectomy	1.23	0.64**–**2.38	0.535			
**Radiotherapy**	**0.29**	**0.16–0.54**	**< 0.001**	**0.116**	**0.02–0.59**	**0.001**
**Chemotherapy**	**3.36**	**1.77–6.37**	**< 0.001**	**7.86**	**1.21–51.24**	**0.031**

*HR* hazard ratio, *95% CI* 95% confidence interval, *BMI* body mass index, *BPH* bad prognostic histology (serous papillary, clear cell, mixed, carcinosarcoma), *LVI* lymphovascular invasion

## Discussion

There is controversy regarding the benefit radical hysterectomy provides to patients with endometrial carcinoma. Retrospective studies have not found a prognostic benefit for this procedure in patients with stage II endometrial carcinoma [8, 9]. Nevertheless, radical hysterectomy could be justified in cases in which the parametrium must be assessed for prognostic purposes and to guide adjuvancy [10]. The National Comprehensive Cancer Network (NCCN) guidelines recommend radical hysterectomy in cases with cervical involvement by image or biopsy to make decisions regarding the type of adjuvancy that will be offered [4]. The European Society for Medical Oncology (ESMO)-European Society of Gynaecological Oncology (ESGO)-European Society for Radiotherapy & Oncology (ESTRO) consensus suggests that the goal of radical hysterectomy involves detecting negative margins and evaluating the type of adjuvancy [11].

In our study, radical hysterectomy did not improve overall survival or disease-free survival in patients with endometrial carcinoma with cervical involvement. The evidence regarding the role of radicality is controversial. In a study where 202 patients with cervical involvement were evaluated, the 5-year overall survival in the radical hysterectomy group was 86%, compared to 61% in the simple hysterectomy group [10]. Likewise, in another study with 203 patients in stage II, the 5-year overall survival rates of patients who underwent radical and simple hysterectomies were 94% and 79%, respectively (*p* < 0.05). However, that study used the 1988 staging criteria, and not all patients received radiotherapy [2]. As to more recent evidence, studies have not suggested a benefit for radical hysterectomy in these patients. In the 2013 GOTIC study, which studied 300 patients with suspected cervical involvement, 74 underwent radical hysterectomy, 112 underwent modified radical hysterectomy, and 114 underwent simple hysterectomy, and no statistically significant difference was found in the 5-year overall survival (83.6% vs. 85.6% vs. 84%) or 5-year disease-free survival (71.6% vs. 77.6% vs. 66.4%). Furthermore, this study found that the only associated factors were age > 55 years, poor prognostic histologies, grade 3 histology, lymph node disease, and ascites or malignant cytology, similar factors to those found in our study [8]. A case-control study from 2016 compared both hysterectomies in patients with type 1 and stage II endometrial carcinoma but found no difference in the 3-year overall survival (88.7% for simple vs. 94.1% for radical, *p* = 0.08) [9]. Likewise, a systematic review published in 2019 found no benefit of radicality for the overall survival (HR 0.92, 95% CI 0.72–1.16) or disease-free survival (HR 0.75, 95% CI 0.39–1.42) of patients with stage II disease [12]. Additionally, our study found that radical hysterectomy is associated with bleeding > 500 ml, longer surgical times, and intraoperative complications, independent of stage. These results are similar to those found in the GOTIC study: higher surgical time, blood loss, need for transfusion, and urinary retention in the radical hysterectomy group [8].

It is important to note that the type of hysterectomy does not appear to alter adjuvant therapy either. In our study, only 2 patients with cervical involvement had parametrial involvement without other factors of poor prognosis, which warranted a change in their adjuvant treatment. Likewise, a study with 334 patients who underwent radical hysterectomy found that only 8.4% of patients had parametrial involvement. Although there was a significant difference in the overall survival and disease-free survival in patients with parametrial involvement in this study, this factor was not proven to be an independent prognostic factor (OR 2.12, 95% CI 0.61–7.81) [6].

Among other findings of our study, a higher percentage of patients who underwent radical hysterectomy had a poor prognostic histology. This finding can be explained by the tumor biology of these histologies, since serous papillary tumors and carcinosarcomas tend to have a greater pelvic spread, which warrants radical surgery to reach optimal cytoreduction without modifying adjuvancy. Furthermore, our study showed that patients with a deeper cervical stromal invasion and a higher ratio of invasion/cervical thickness tended to be those who underwent radical surgeries, probably because they are easier to identify clinically and are prepared to undergo radical surgery from the start. It is also interesting to point out that para-aortic lymphadenectomy is more common in radical hysterectomy, since this procedure is more frequent in patients with poor prognostic histologies and higher grades.

The main weaknesses of our study are its retrospective nature, the low number of patients who underwent radical hysterectomy compared to simple hysterectomy, the median follow-up time, and the inclusion of multiple histologies. However, this is the first study to evaluate the outcome of these procedures in a Latin American population.

## Conclusions

In patients with endometrial carcinoma with cervical involvement, radical hysterectomy does not improve prognosis or alter adjuvant therapy. However, it is associated with a longer surgical time, risk of intraoperative complications, and bleeding of over 500 ml. Prospective studies are needed to better elucidate the role of radical hysterectomy in Latin American patients with endometrial carcinoma.

## Data Availability

The datasets generated and/or analyzed during the current study are not publicly available due to hospital policy but are available from the corresponding author on reasonable request.

## Abbreviations

IQR: Interquartile range
OR: Odds ratio
CI: Confidence interval
SH: Simple hysterectomy
RH: Radical hysterectomy
OS: Overall survival
DFS: Disease-free survival
HR: Hazard ratio
